# Trends and factors associated with retention in HIV care among men living with HIV in a peri-urban primary care facility in central Uganda: a retrospective cohort study

**DOI:** 10.1186/s12889-025-23161-w

**Published:** 2025-05-21

**Authors:** Victoria Babirye Tumusiime, Joan Nangendo, Anthony Kirabira, Moses Mugerwa, Jonathan Mayito, Aggrey David Mukose, David Kyaddondo

**Affiliations:** 1 School of Public Health, College of Health Sciences, Makerere University, Kampala, Uganda; 2 MRC/UVRI/LSHTM Uganda Research Unit, Entebbe, Uganda; 3 Clinical Epidemiology Unit, School of Medicine, Makerere University College of Health Sciences, Kampala, Uganda; 4 Infectious Diseases Research Collaboration, Kampala, Uganda; 5 Baylor Foundation, Kampala, Uganda; 6 Infectious Diseases Institute, College of Health Sciences, Makerere University, Kampala, Uganda; 7 Child Health and Development Center (CHDC), College of Health Sciences, Makerere University, Kampala, Uganda

**Keywords:** Men living with HIV, Sub-Saharan Africa, Trends, Retention in HIV care

## Abstract

**Introduction:**

Research on retention in care among men living with HIV (MLHIV) in Sub Saharan Africa is limited. This study examined trends and factors associated with retention in HIV care among men in Wakiso district, Uganda, to identify potential targets for interventions aimed at improving retention.

**Methods:**

We retrospectively analyzed 833 electronic records of MLHIV who were initiated on ART at Wakiso Health Centre IV between January 2018 and December 2021 in two cohorts, MLHIV initiated on ART (January 2018 to December 2019) pre-coronavirus disease 2019 (COVID-19) and (January 2020 to December 2021) during the COVID-19 pandemic. The trends of retention in HIV care were graphically assessed using line plots. A mixed effects modified Poisson model was used to assess factors associated with retention in care.

**Results:**

The prevalence of retention in care at 6 months was high (62.9%) pre-COVID-19 and 71.4% during COVID-19 and dropped to below 50% in both cohorts by 24 months. Factors associated with retention in care were ownership of a mobile phone (aPR: 1.10; 95%CI: 1.05–1.28) and (aPR: 1.24; 95%CI: 1.13–1.43). Advanced disease (aPR: 0.76; 95%CI: 0.61–0.94) and (aPR: 0.68; 95%CI: 0.47–0.96) was associated with a lower prevalence of retention. Facility-based groups (aPR: 1.12; 95%CI: 1.02–1.24) were associated with a high prevalence of retention, while facility-based individual management (aPR: 0.91; 95%CI: 0.83–0.99) was associated with a lower prevalence of retention compared to community drug distribution points (CDDP). Multi-month dispensing of over 3–5 months (aPR: 1.51; 95%CI: 1.20–1.90) and 6-months pills (aPR: 1.49; 95%CI: 1.18–1.88) compared to 1-month dispensing was significantly associated with a high prevalence of retention.

**Conclusion:**

The trend of retention in HIV care among MLHIV in this study declined with increasing duration on ART and may require tailored interventions for men to be retained on lifelong ART. Multi-month dispensing of ART, patients’ mobile phones and facility-based groups had a positive influence on retention in care among MLHIV and may be further explored as possible interventions to increase retention in this population.

## Background

Globally, over 39.9 million people were living with HIV, and while 30.7 million were accessing ART in 2023. The worldwide scale-up of antiretroviral therapy (ART) has dramatically improved outcomes for people living with HIV (PLHIV), enabling viral suppression and a near-normal life expectancy [1]. Sustained retention in care is crucial for realizing these benefits, as it ensures consistent ART adherence, viral suppression, and ultimately, improved quality of life [2–4]. Despite increased access to ART, a significant challenge remains in maintaining long-term retention, particularly among men [5, 6]. Studies consistently identify male gender as a risk factor for loss to follow-up across diverse settings [7, 8].

In Uganda, while research on HIV care has advanced, studies have predominantly focused on the general PLHIV population, pregnant women, and the youth [9, 10]. Although innovations such as multi-month dispensing, differentiated service delivery models (DSDM) have been observed to increase retention among these groups [11] Focusing on only those groups has left a critical gap in understanding the specific challenges faced by men living with HIV (MLHIV) in Uganda. Men may experience unique barriers to engagement in care potentially related to gender norms, occupational demands, or healthcare access preferences [12, 13]. For example, masculine ideals of self-reliance and aversion to perceived weakness may discourage men from seeking or adhering to healthcare services [14, 15]. Furthermore, the limited research specifically targeting MLHIV in Uganda hinders the development of tailored interventions to address their distinct needs. This study addresses this critical gap by examining the trends and factors associated with retention in HIV care specifically among MLHIV in Wakiso district. By focusing on this understudied population, this research aims to identify key drivers of retention and inform the design of targeted interventions to improve long-term retention in care and ultimately, health outcomes for MLHIV in Uganda.

## Methods

### Study design

We carried out a retrospective cohort study involving secondary analysis of routinely collected data of MLHIV aged 18years and above. Data were collected from January 2018 to December 2021 in two cohorts. January 2018 to December 2019 pre COVID 19 cohort and January 2020 to December 2021 during COVID19 cohort.

### Study setting

The study was conducted at Wakiso Health Center IV located in Wakiso district in Central Uganda. Wakiso is one of the largest districts in the country and surrounds Kampala, the capital city of Uganda. It also serves as a residential area for many people who work in the city. Wakiso district notably has a high HIV prevalence rate of 10% which is nearly double the national adult average of 5.1% [16]. The district has 66 public and private health facilities that actively provide ART services [17]. For this study, we purposively selected the largest centrally located public health facility. This facility operates a comprehensive HIV care clinic with a wide catchment area and serves a population comparable to that of other facilities within in Wakiso district. It is primarily supported by the government of Uganda with additional funding from implementing partners to offer cost-free services. The facility delivers integrated HIV services supported by counsellors and village health teams who play a critical role in promoting retention and adherence through counselling and client follow up.

### Study population

The study population was MLHIV initiated on ART at Wakiso Health Center IV between January 2018 and December 2021. We included de-identified medical records of MLHIV aged 18 years and above, who had been initiated on ART at Wakiso Health Centre IV. MLHIV medical records were excluded if they missed important variables such as date of starting ART, age. A total of 848 records were extracted from the OpenMRS database during the study period and screened. However, only 833 records were included in the final analysis, as 15 records lacked data on either the patient’s age or the date of ART initiation the key variables required for analysis.

### Study outcomes and variables

Retention in HIV care was the primary study outcome. MLHIV were considered retained at 6, 12, 18, and 24 months post-ART initiation if they had at least one visit, refill, or viral load test within a 90-day window around each of these time points excluding those recorded as deaths, transferred out and lost to follow up [18]. The independent variables were extracted from the open-source medical records system (OpenMRS) and included year of ART initiation, age, advanced HIV disease status, ARV regimen, Body Mass Index (BMI), World Health Organization (WHO) stage, tuberculosis (TB) status, and ownership of a mobile phone. In addition, the service delivery factors such as DSDM, specifically, if a MLHIV was enrolled in a facility-based group, and facility based individual management or a community drug distribution point. The ART dispensing interval was also assessed indicating, whether participants were prescribed ART for 1, 2, or 3 or6 months.

### Data collection

Anonymized patient data of MLHIV > 18 years of age initiated on ART at Wakiso Health Centre IV were extracted from the OpenMRS database into Microsoft Excel Version 16.0. OpenMRS is a system used to routinely manage patients in all public health facilities in Uganda. Briefly, data in the OpenMRS is extracted from routine ART clinic cards used by clinicians and entered into the system by a data officer at each health facility. Study data was extracted in June 2023 with the help of data managers at the facility. The data extracted included demographics, and routine clinical management data (which were collected as the patients were managed in the clinic).

### Data analysis

We imported the data from the Excel Version 6.0 into Stata Version 17.0 (Stata-Corp College Station, TX, USA) for analysis. Retention was assessed as a binary outcome categorised as retained or not retained. Records of men who were transferred out or died or were lost follow up were treated as retained until the date of the event (lost to follow up, death or transfer out), after which they were excluded from the analysis. We assessed retention in HIV care using six monthly intervals at 6, 12, 18, and 24 months. We used the 6-month intervals to keep in line with the multi-month prescriptions guidance that is currently used to differentiate services for stable clients in Ugandan public health facilities.

Descriptive statistics of the demographics and clinical characteristics used proportions and frequencies for categorical variables. For bivariate analysis, we used chi-square and Fischer’s exact tests as an alternative in instances of sparse data to determine association and significance of retention in care against the predictor variables. For multivariable analysis, we used a forward stepwise approach to mixed effects modified Poisson regression and adjusted for the repeated measures of retention at 6, 12, 18, and 24 months. A *p*-value ≤ 0.25 was considered significant for selection of variables into the model, we included known confounders such as age, in the model regardless of their *p*-value, based on prior evidence of their association with retention in care. We eliminated variables with *p*-values > 0.05 from the model and retained those with *p*-values < 0.05 until model parsimony was achieved. The effect measure was reported as adjusted prevalence ratio (aPR) with its corresponding 95% confidence interval (95%CI).

We analyzed factors associated with retention in HIV care separately for MLHIV initiating ART between January 2018 and December 2019 (pre-COVID-19) and January 2020 and December 2021 (during COVID-19), resulting in two distinct models. We segmented the data to account for potential changes in treatment protocols or external factors over time, such as disruptions caused by COVID-19.

## Results

### Description of study participants

A total of 833 records of MLHIV who were initiated on ART between January 2018 and December 2021 were included in the analysis. Of these, 455 were initiated between January 2018 and December 2019 (pre-COVID-19), and 378 were initiated between January 2020 and December 2021 (during the COVID-19 pandemic). Many of the MLHIV were between 35 and 49 years of age (48.8%, 222/455) pre-COVID-19 and (43.4%, 164/3780) during COVID-19.

The majority of MLHIV had a normal BMI (72.6%, 168/455) pre-COVID-19 and (66.6%, 219/378) during COVID-19, and were initiated on the TDF/3TC/EFV regimen (52.1%, 233/445) pre-COVID-19 and (97.4%, 368/378) during COVID-19. The majority of MLHIV were enrolled in facility-based individual management (FBIM) (42.3%, 118/455) pre-COVID-19 and (61.8%, 218/378) during COVID-19.

More men in the pre-COVID-19 cohort (40.0%, 182/455) were dispensed pills that lasted 1 month, while in the during COVID-19 cohort, more men were dispensed pills that lasted 3–5 months (48%,182/3780). The baseline characteristics of the participants who initiated ART in the two cohorts (pre-COVID-19 and during COVID-19) are shown in Table 1.

**Table 1:** Baseline characteristics of the participants who initiated ART in pre-COVID-19 and during COVID-19

Variable	Pre-COVID-19 (*N* = 455)	During COVID-19 (*N* = 378)	Chi-square/Fisher’s exact
	*n* (%)	*n* (%)	*P*-value
**Age group (years)**			
18–24 25–34 35–49 ≥ 50	9 (2.0) 149 (32.8) 222 (48.8) 75 (16.5)	16 (4.2) 143 (37.8) 164 (43.4) 55 (14.6)	0.095
Advanced HIV disease*			
No Yes	233 (99.6) 1 (0.4)	261 (96.7) 9 (3.3)	**0.020**
**Viral load**			
Undetectable *≥* 50 copies/ml	273 (92.5) 22 (7.5)	243 (92.8) 19 (7.2)	0.926
**Differentiated service delivery models**			
Community drug distribution point Facility-based groups Facility-based individual management Fast-track drug refill	53 (19.0) 23 (8.2) 118 (42.3) 85 (30.5)	43 (12.2) 15 (4.25) 218 (61.8) 77 (21.8)	**< 0.001**
**ARV regimen at ART initiation**			
TDF/3TC/DTG TDF/3TC/EFV	214 (47.9) 233 (52.1)	368 (97.4) 10 (2.7)	**< 0.001**
**ARV pills dispensing interval and duration of coverage**			
1 month 2 months 3–5 months ≥ 6 months	182 (40.0) 40 (8.8) 168 (36.9) 65 (14. 3)	111 (29.4) 14 (3.7) 182 (48.3) 70 (18.6)	**< 0.001**
**BMI**			
Underweight (< 18.5) Normal weight (18.5–24.9) Overweight (25–29.9) Obese (≥ 30)	27 (11.95) 164 (72.6) 25 (11.1) 10 (4.4)	22 (6.7) 219 (66.6) 74 (22.5) 14 (4.26)	**0.002**
**WHO stage**			
Stage 1 Stage 2 Stage 3	300 (97.1) 3 (0.9) 6 (1.9)	236 (94.4) 3 (1.2) 11 (4.4)	0.233
**TB status**			
Diagnosed with TB/receiving TB treatment No symptoms of TB	13 (2.8) 441 (97.1)	15 (4.1) 354 (95.9)	0.344
**Ownership of a mobile phone**			
No Yes	83 (18.2) 372 (81.8)	35 (9.3) 343 (90.7)	**< 0.001**

*Defined as CD4 cell count < 200 cells/mm3

### Trend of retention in care at 6, 12, 18 and 24 months among MLHIV who initiated ART between January 2018 and December 2019 pre-COVID-19 and among MLHIV who initiated ART between January 2020 and December 2021 during COVID-19

**Fig. 1:**
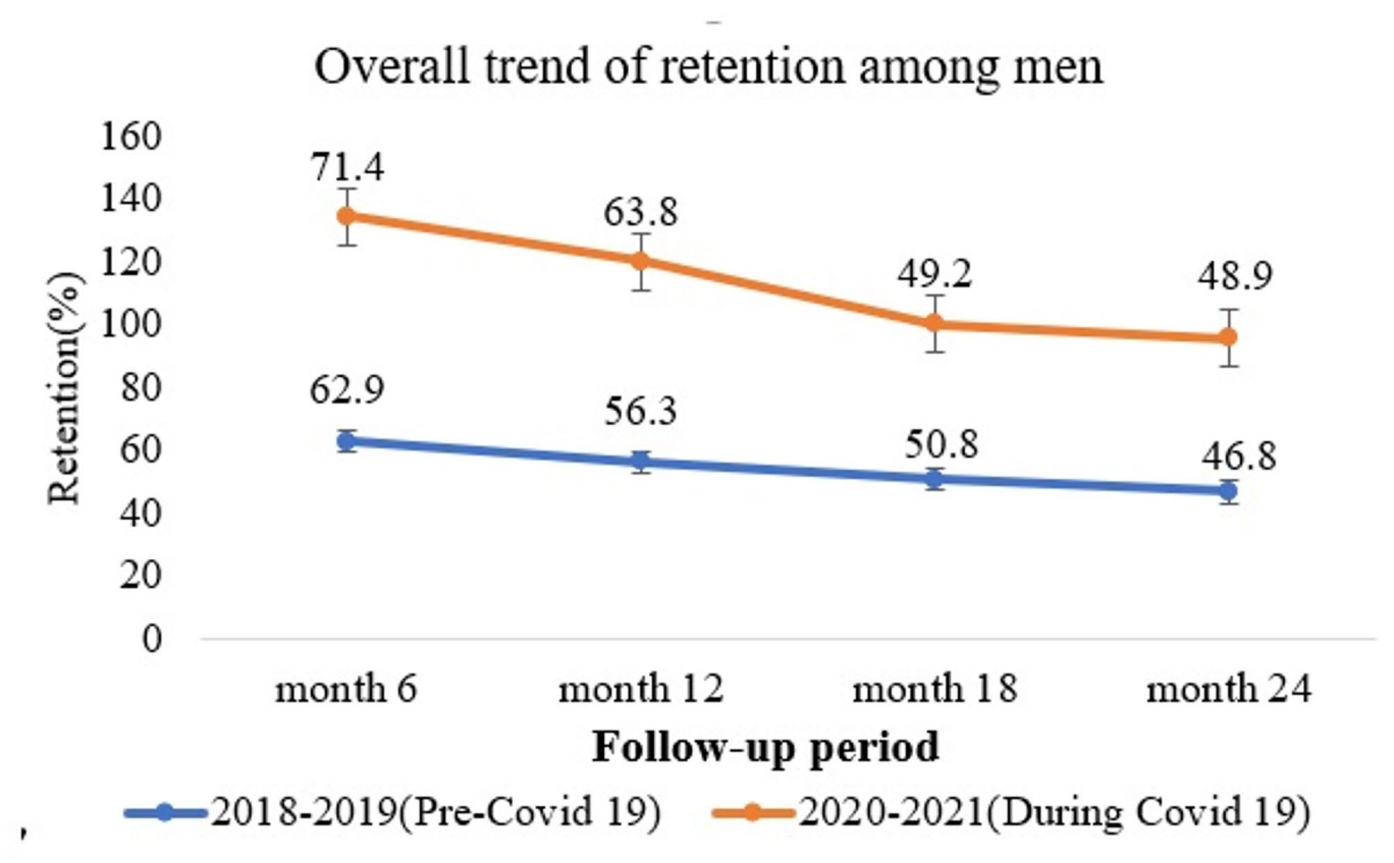
Trends in retention of MLHIV initiated in the pre and during the COVID-19 cohort and the respective error bars at 95%CI

Retention in HIV care rates declined over time in both cohorts, particularly from 6 months to 24 months. The highest retention in both periods was at 6 months, at 62.9% (286/455) in pre-COVID-9 cohort and 71.4% (270/378) in the during COVID-19 cohort. The lowest was at 24 months, 46.8% (213/445) in the pre-COVID-19 cohort and 48.9% (158/378) in the during COVID-19 cohort. Overall, the during COVID-19 cohort showed higher prevalence of retention compared to the pre-COVID-19 cohort (Fig. 1).

### Factors associated with retention in HIV care among MLHIV who initiated ART pre-COVID-19 and during COVID-19

Adjusted analysis suggested the following factors associated with retention in care among MLHIV.

*Pre-COVID-19 Cohort*: In this cohort, while adjusting for other factors, mobile phone ownership (aPR: 1.10; 95%CI: 1.05–1.28) was associated with a higher prevalence of retention in care compared to those with no mobile phone.

Furthermore, MLHIV receiving care under facility-based groups were significantly associated with a higher prevalence of retention of care (aPR: 1.12; 95%CI: 1.02–1.24) compared to MLHIV receiving care under community drug distribution points while adjusting for other factors.

In addition, MLHIV initiated on ART in WHO stage 3 (aPR: 1.15; 95%CI: 1.02-–1.32) were significantly associated with higher prevalence of retention compared to those in WHO stage 1, while MLHIV with advanced disease (aPR: 0.76; 95%CI: 0.61–0.94) were significantly associated with lower prevalence of retention compared to those with no advanced disease.

*During COVID-19 Cohort*: In this cohort, while adjusting for other factors, mobile phone ownership (aPR: 1.24; 95%CI: 1.13–1.43) compared to those without mobile phones was significantly associated with a higher prevalence of retention and MLHIV who were given multi-month dispensing (aPR: 1.51; 95%CI: 1.20–1.90) for 3–5 months; (aPR: 1.49; 95%CI: 1.18–1.88) for 6 months compared to those dispensed 1-month supply of pills were associated with a significantly higher prevalence of retention.

MLHIV receiving care under facility based individual management (aPR 0.91; 95%CI: 0.83–0.99) were associated with a lower prevalence of retention compared to MLHIV under community drug distribution points while adjusting for other factors.

In addition, MLHIV initiated on ART with advanced disease (aPR: 0.68; 95%CI: 0.47–0.97) were significantly associated with a lower prevalence of retention as well as those who TB history at baseline (aPR: 0.53; 95%CI: 0.29–0.96) compared to those without TB.

In this cohort, the prevalence of retention differed by age. The prevalence of retention among MLHIV in the age groups 25–34 (aPR: 0.83; 95%CI: 0.73–0.96) and 35–49 (aPR: 0.86; 95%CI: 0.76–0.98) was significantly lower than that of those aged 18–24 years while adjusting for other factors as shown in Table 2.

**Table 2:** Factors associated with retention among MLHIV who initiated ART between 2018 and 2021 in two cohorts at Wakiso health centre IV

Variable	Pre COVID-19		During COVID-19	
	Crude PR (95% CI)	Adjusted PR (95% CI)	Crude PR (95% CI)	**Adjusted PR (95% CI)**
**Age group (years)**				
18–24 25–35 35–49 ≥ 50	1 1.11 (0.58–2.12) 1.15 (0.60–2.19) 1.28 (0.67–2.47)	1 1.26 (0.97–1.65) 1.17 (0.90–1.52) 1.12 (0.84–1.49)	1 1.02 (0.69–1.50) 1.13 (0.77–1.65) 1.06 (0.71–1.59)	1 **0.83 (0.73**–0.96) ** 0.86 (0.76–0.98) * 0.88 (0.76–1.02)
**Advanced HIV disease†**				
No Yes	1 0.91 (0.88–0.96) ***	1 **0.76 (0.61**–0.94) *	1 0.48 (0.32–0.72) ***	1 **0.68 (0.47**–0.97) *
**Viral load**				
Undetectable > 50 copies/ml	1 0.87 (0.72–1.06)		1 0.88 (0.71–1.08)	
**ARV pills dispensing interval and duration of coverage**				
1 month 2 months 3–5 months ≥ 6 months	1 2.00 (1.48–2.74) *** 3.89 (3.09–4.90) *** 3.84 (3.02–4.88) ***	1 0.85 (0.66–0.08) 0.94 (0.81–1.08) 0.92 (0.77–1.08)	1 1.49 (0.72–3.06) 4.05 (3.10–5.29) *** 4.14 (3.15–5.44) ***	1 1.21 (0.56–2.61) 1.51 (1.20–1.90) *** 1.49 (1.18–1.88) **
**Differentiated service delivery models**				
Community drug distribution Facility-based groups Facility-based individual Fast-track drug refill	1 0.97 (0.82–1.14) 0.81 (0.73–0.90) *** 0.95 (0.87–1.03)	1 1.12 (1.02–1.24) ** 0.92 (0.83–1.04) 0.96 (0.87–1.06)	1 0.94 (0.80–1.11) 0.59 (0.53–0.66) *** 1.01 (0.94–1.09)	1 0.97 (0.82–1.14) 0.91 (0.83–0.99) * 1.05 (0.96–1.13)
**ARV regimen at ART initiation**				
TDF/3TC/DTG TDF/3TC/EFV	1 0.88 (0.76–1.02)		1 1.14 (0.82–1.57)	
**BMI**				
Underweight (< 18.5) Normal weight (18.5–24.9) Overweight (25–29.9) Obese (≥ 30)	1 1.14 (1.03–1.26) * 1.04 (0.90–1.19) 0.90 (0.66–1.21)		1 0.82 (0.63–1.06) 1.05 (0.93–1.18) 1.02 (0.78–1.35)	
**WHO stage**				
Stage 1 Stage 2 Stage 3	1 0.06 (0.03–0.09) *** 0.99 (0.72–1.38)	1 0.96 (0.86–1.06) 1.15 (1.02–1.32) *	1 1.10 (0.64–1.91) 0.77 (0.62–0.96) *	
**TB status**				
No TB TB history	1 0.34 (0.14–0.87) *		1 0.25 (0.12–0.52) ***	1 0.53 (0.29–0.96) *
**Ownership of a mobile phone**				
No Yes	1 1.02 (0.93–1.39)	1 1.10 (1.05–1.28) *	1 0.84 (0.74–1.16)	1 1.24 (1.13–1.43) *

Secondary data: PR = Prevalence Ratios; *Statistically significant at p < 0.05 *,p < 0.01 **,p < 0.001 ***. Variables indicated in bold had a *P*-value of < 0.05 and hence showed a statistical association with the outcome variable. **†**Defined as CD4 cell count < 200 cells/mm3

## Discussion

This study assessed the trends and factors associated with retention in HIV care of MLHIV at 6, 12, 18, and 24 months during pre-COVID-19 and during the COVID-19 period in a peri-urban public health facility in Wakiso district, Uganda. It was also found that retention in HIV care declined over time, dropping to below 50% at 24 months after ART initiation. We observed a declining trend of prevalence of retention in HIV care similar to that observed in another study among men in South Africa [19]. This observed decline in retention rate with duration from ART initiation could be related to factors such as treatment fatigue, stigma or even competing life activities. While there is not a lot of data among MLHIV, other PHIV populations such as adolescents [10], and pregnant and breastfeeding mothers on ART [20] have also exhibited the same trend. Other studies have also indicated a declining trend in retention from ART initiation, but a higher retention rate after two years. A study in Kinshasha, Democratic Republic of the Congo, showed a decline from 83% at 12 months to 77% at 24 months but among PLHIV [21]. This may, in part, be due to the studies using mixed adult populations which could give the advantage of presence of women who are known to be better retained than men [5]. Notable barriers against retention in care have been previously identified as lack of transport facilities [22], long waiting time and masculine attitudes [23, 24], which shape men’s continued access to care. Although these factors were not directly assessed in this study, the observed higher prevalence of retention among MLHIV receiving services that reduced the travel frequency and time spent at the facility such as facility-based groups, and multi-month dispensing suggests a potential link to long waiting time and transport difficulties. Furthermore, despite the ART clinic’s efforts, the unexpectedly low retention among MLHIV in this study could indicate the influence of masculine attitudes. These findings warrant further investigation to explore these potential associations more directly. Therefore, while not directly measured, our findings offer suggestive evidence pointing towards the potential impact of long waiting times, transport difficulties and masculinity attitudes on retention in HIV care of MLHIV.

The prevalence of retention in HIV care among MLHIV initiated in the during COVID-19 period was higher compared to the pre-COVID-19 period, despite the inherent interruptions in the health systems. Although the differences in the trends in the cohorts may not have been statistically significant, it was still informative from a pragamatic point of view. It could have been that the interventions used during the COVID-19 pandemic were more effective and convenient for the MLHIV. During the COVID-19, HIV care in health facilities relied on community-based modes of delivery of care [25]. Additionally, due to the COVID-19 restrictions (e.g., lockdowns), people were not working, long-distance travel for work was restricted, and leisure activities that men typically engaged in were limited. As a result, MLHIV were more available to attend to their care. Furthermore, the health-related messages regarding the effect of HIV on prognosis of COVID-19 might also have motivated the men to remain in HIV care. The difference in trends in both cohorts was not statistically modelled and therefore could be further explored with the possibility that the service delivery models used during COVID-19 could have been suitable for MLHIV who already face constraints regarding retention in care.

Retention in care was significantly associated with other factors in this study. The prevalence of retention among MLHIV who owned a mobile phone was higher than those who did not own one in both cohorts. Mobile phones are key tools for enhancing retention because they aid in reminder strategies, patient follow-up and continuous counseling. This could explain why those who had mobile phones had a higher prevalence of retention. These findings are comparable to those obtained from a study conducted in the Wakiso district which examined loss to follow-up, and indicated that PLHIV who did not own mobile phones were significantly more likely to be non-retained [9]. Similarly, another study on determinants of lost to follow up found that the patients without a mobile phone were 52% more likely to get lost to follow-up [26]. Overall, this study finding concurs with the findings in literature.

DSDM also influenced retention in HIV care in both pre-COVID-19 and during COVID-19 differently. The DSDM offers MLHIV various ways to provide care to improve individual outcomes by considering their preferences and aims at clinic efficiency by reducing congestion at the ART clinics. The major DSDM used in Uganda include those that aim at accessing care in the community such as community drug distribution point, community pharmacies and community client led ART and facility-based models such as fast-track drug refills, facility-based groups and facility-based individual management [11]. Facility-based groups enable MLHIV to form groups and rotate in picking up their ART from the facility, where they also receive health worker assessments. This allows other men in the group to concentrate on their work until it is their turn to visit the facility. Among the pre-COVID-19 cohort, MLHIV receiving care in the facility-based groups were more retained compared to those in the community drug distribution while among the during COVID-19 cohort, MLHIV receiving care in the facility based individual management was associated with lower prevalence of retention in HIV care. This could have been probably due to restrictions in movement during the COVID-19 pandemic but also the facility individual based management requires a lot of time and is prone to stigma. This, however, depicts the changing nature of MLHIV’s preferences in receiving care and these should be considered as new HIV delivery interventions are designed. As it was noted that no single DSDM is likely to meet the needs and preferences of all individuals receiving HIV care, countries must decide which combination of DSDM is the best fit for their setting [27].

We observed significantly higher retention in multi-month dispensing among MLHIV initiated during the COVID-19 pandemic. MLHIV dispensed with 3–5 months and 6 months of pills had higher retention rates compared to those receiving only 1 month’s supply. This could be due to the flexibility it gives men to benefit from care without compromising their competing economic and leisure activities. Similarly, a clinical trial carried out in Malawi and Zambia found that dispensing ART every 6 months was non-inferior to 3-month dispensing and concluded that multi-month dispensing could be a strategy that improves retention in care in resource-constrained countries [28]. Another study, carried out in Haiti, found that extending ART dispensing intervals improved retention 12 months after ART initiation [29]. Another quantitative study found that increasing dispensing intervals was associated with high retention at 12 months [29]. This is suggestive of a potential strategy to further explore for improvement of retention in HIV care among MLHIV.

Advanced HIV disease was associated with lower retention in care in our study. MLHV initiated on ART in both cohorts who had advanced HIV disease were less likely to have been retained in HIV care compared to men without advanced HIV disease. Whereas some men might have died due to the condition of having advanced disease, others may have simply been lost to follow up. These findings are consistent with other studies that indicated that advanced HIV disease was associated with attrition and loss to follow up [21, 30]. Men who were in WHO clinical stage 3 at baseline had a significantly high retention in care compared to MLHIV in WHO stage 1 in the pre-COVID-19. Contrary to these findings, studies have found that participants in WHO stage 3 and above were less likely to be retained in care [10, 21, 26]. However, there have been qualitative studies that indicated that MLHIV fear to be associated with disease or ill health [6] and they believe that not attending care makes one look sick and hence a motivator to stay in care [24]. Among MLHIV initiated on ART during COVID-19, TB status at baseline was associated with a lower rate of retention in care. Prior studies have similarly shown that patients on TB treatment or those who had a history of TB treatment were significantly more likely to be lost to follow up [31], as were those on both ART and TB treatment [32]. The low retention in HIV care among the ART and TB patients could probably be due to the higher pills burden [33] but it may also indicate that some succumbed to TB or COVID-19 since they have a similar clinical presentation.

In this study, MLHIV in the age groups of 25–35 years and 35–49 years had lower rates of retention in care compared to participants in the age group 18–24 years. Other studies found age to be significantly associated with retention in care [7, 21, 34], with retention being lower among the younger age groups (18–24 years). In a study done in Masaka district, Uganda, age greater than 25 years was associated with decreased loss to follow up rates [26]. In another study among PLHIV in Wakiso district, Uganda, patients less than 30 years of age had a higher incidence of lost to follow up [9]. Over time, many studies and interventions have majorly focused on the youth. Under Consolidated guidelines for the prevention and treatment of HIV and AIDS in Uganda the youth have a special program called young people and adolescent peer support program (YAPS) that has enhanced their retention in care [35]. This could partly explain the difference in findings among MLHIV ages in our study and strengthens the argument that MLHIV also need special interventions for retention in HIV care. However, MLHIV in this age group are most likely burdened with social economic factors, stigma and competing life activities which could additionally affect retention in care. A more accurate understanding will require additional qualitative investigation.

This study has several limitations. Given that the original data was not collected specifically for the research questions addressed in this study means that the findings may not be generalizable to populations or settings outside of the context of this study. As an additional limitation with utilization of routinely collected data from HIV programs, a common but complex data source often affected by high levels of missing information, the data in this study had up to 30% missingness in some variables. But overall, the data contained over 3000 observations which was adequate to give us valid results despite the missingness. Due to the limited amount of data available in-patient records in OpenMRS, we are unable to further investigate the differences in the prevalence of MLHIV retention to ascertain the observed variations. In addition, the classification of some participants as non-retained may be inaccurate, as they may have transferred to other facilities or died. Consequently, this could lead to a misclassification of lost to follow-up and an underestimation of retention in care in this study. A study on correcting retention estimates among PLHIV suggests that relying solely on routine clinic data can inflate the estimation of lost to follow up [22], a potential issue in our study. Therefore, the reported retention rates should be interpreted considering this limitation. Correction of these estimates was beyond the scope of this study; future research could explore this further.

The study was carried out in a peri Urban facility and may only describe similar settings, future studies should be done across different facilities in different regions. Furthermore, reasons for disengagement were not assessed in this study, hence the need to assess the barriers and facilitators of retention in care among MLHIV in future studies. However, the results of this study provide insights for further investigations into the significant factors associated with retention in care among MLHIV.

## Conclusion

The prevalence of retention in HIV care among MLHIV observed in this study was concerningly low and underscores the possibility of achieving optimal treatment outcomes and epidemic control. This low retention was significantly associated with advanced HIV disease, tuberculosis at baseline, and WHO stage 3. The study’s findings highlight the urgent need for more targeted interventions to improve retention among this population. Optimising multi-month dispensing (e.g. providing 3–6-month supplies based on adherence history and stability)., refining differentiated service delivery models to align with diverse preferences and circumstances of MLHIV such as facility-based groups, community drug distribution points and options leveraging effective use of mobile phones for appointment reminders, and follow up could improve retention among MLHIV. The lessons learned from analysis of the during COVID-19 cohort in this study particularly support the possible effectiveness of multi-month dispensing, and that community-based approaches such as community drug distribution point and community health worker support for retention should inform future efforts to enhance retention. Further research is needed to explore barriers and facilitators to retention experienced by different subgroups of MLHIV (e.g. the young adults 25–35 and middle aged 35–49, those with co-morbidities like TB and those with advanced HIV disease) to inform the development of tailored and effective interventions. Addressing low retention rates among MLHIV in Uganda requires urgent and focused attention on implementing and rigorously evaluating this tailored intervention to improve health outcomes and ultimately contribute to achieving national HIV epidemic control goals.,

## Data Availability

Data are available on the Zenodo database at DOI https://doi.org/10.5281/zenodo.14859078. This data is held under Creative Commons Attribution 4.0 International (CC-BY 4.0).

## Abbreviations

ADHD: Advanced Hiv disease
ART: Antiretroviral therapy
BMI: Body mass index
COVID-19: Coronavirus disease 2019 pandemic
DSDM: Differentiated service delivery model
HIV: Human immunodeficiency virus
OpenMRS: Open medical resource system
UNAIDS: Joint united nations programme on HIV/AIDS
WHO: World health organization
YAPS: Young people and adolescent peer support
